# Spiritual Support for People Affected by Dementia: A Scoping Review

**DOI:** 10.1177/14713012251377812

**Published:** 2025-09-09

**Authors:** Emma Wolverson, Tamara Backhouse, Alice Burnand, Siren Eriksen, Andrea Fonseca de Paiva, Karen Harrison Dening, Jean-Bernard Mabire, Serena Sabatini, Jenny T. van der Steen

**Affiliations:** 1 Research and Publications, Dementia UK, London, UK; 2 The Geller Institute of Ageing and Memory, 7364 The University of West London, London, UK; 3 School of Health Sciences, 6106 University of East Anglia, Norwich, UK; 4 155319 Lovisenberg Diaconal University College, Oslo, Norway; 5 School of Health Sciences, 3660 University of Surrey, Surrey, UK; 6 Health and Life Sciences, De Montfort University, Leicester, UK; 7 PAVeA Laboratory, Department of Psychology, 27092 University of Tours, Tours, France; 8 Department of Clinical Psychology and Psychobiology, 16724 University of Barcelona, Barcelona, Spain; 9 School of Psychology, 3660 University of Surrey, Guildford, UK; 10 Department of Public Health and Primary Care, 4501 Leiden University Medical Center, Leiden, The Netherlands; 11 Department of Primary and Community Care, 6034 Radboud University Medical Center, Nijmegen, The Netherlands

**Keywords:** dementia, spirituality, psychosocial intervention, spiritual support, religious and spiritual coping, palliative care

## Abstract

As a life-limiting illness, dementia requires a holistic approach to care, where spiritual support plays a crucial role in helping individuals and their caregivers find meaning and solace. Our aim was to systematically map the research conducted on psychosocial interventions developed to provide spiritual support for people living with dementia and their caregivers from diagnosis and across the disease trajectory. A scoping review was conducted to explore the breadth of research on ‘spiritual support’ in dementia care, encompassing interventions, service delivery models, programs, toolkits, approaches, and activities. Electronic databases (MEDLINE (Pubmed), CINAHL, PsycINFO, EMBASE, Web of Science and The Cochrane Library) from inception until February 2025. References of included papers were hand searched. The quality of studies was assessed using the Mixed Methods Appraisal Tool. The findings were interpreted jointly with seven people with dementia and six family caregivers. Twelve papers met the eligibility criteria, reporting on interventions for people with dementia, caregivers, staff skills, care environments, and inclusive worship. Most studies were exploratory, with only one RCT. Studies originated from the USA (n = 4), Europe (n = 4), Australia (n = 2), Taiwan (n = 1), and Canada (n = 1). Eight focused on community settings, three on residential care, and one included both. Six studies involved people with dementia: four with mild to moderate, one with moderate to advanced, and one with mixed severity. Outcomes were inconsistent and there was a lack of longitudinal observational studies to track changes over time. Spiritual support should be personalised and multifaceted, incorporating creative activities and tailored interventions that reflect individual preferences and diverse backgrounds. Future research should employ longitudinal observational and intervention designs.

## Introduction

Receiving a diagnosis of dementia, a life-limiting and progressive illness, is a significant life event for both the individual and their family. National Dementia Strategies across Europe have encouraged timely diagnosis to facilitate future care planning (Alzheimer Europe, 2021). However, the availability of psychosocial support following diagnosis varies both within and across countries (World Health Organisation, 2012). This form of support has primarily focused on maintaining cognition, improving quality of life, and educating caregivers to enhance their coping skills (Hui et al., 2021; McDermott et al., 2019).

One notable criticism of psychosocial interventions in dementia care is their frequent neglect of spiritual components (Daly et al., 2019). Indeed, the term ‘psychosocial’ does not include spiritual. While spiritual support is often considered distinct from psychological and social care within palliative care frameworks, we view it as closely interconnected. Spiritual support addresses emotional, existential, and relational needs, which are deeply intertwined with psychological and social well-being. In this sense, spiritual care complements and enhances psychosocial support by contributing to the holistic well-being of individuals. Relative to psychosocial interventions, spiritual support has been somewhat neglected in dementia research, meaning that spiritual needs that exist may not be addressed. Indeed, Britt et al. (2023) conclude that spiritual needs are minimally addressed in dementia care.

The neglect of spiritual support may be the result of several factors, including the challenge of needing to understand the individual, which becomes more difficult as dementia progresses. Additionally, practitioners often lack the knowledge, skills, and experience to effectively address these needs (Daly et al., 2019). Given that spiritual care is regarded as an essential component of palliative care (World Health Organisation, 2002), the failure to recognize dementia as a progressive and life-limiting condition may also be a factor.

Yet, research indicates that addressing the spiritual needs of people living with dementia is crucial for overall wellbeing. Reviews (Agli et al., 2015; Britt et al., 2023; Daly et al., 2019) highlight the importance of spirituality for people with dementia in discovering purpose, maintaining hope, and establishing connections with one’s past, present, and future, as well as managing the condition. If spiritual needs and concerns are not addressed, spiritual distress may occur, which is understood as impaired ability to connect with others and derive less meaning from life (Caldeira et al., 2013). Unmet spiritual needs have been linked to poorer emotional and spiritual well-being, increased psychological distress, and reduced quality of life in adults with serious illness, including cancer (Delgado-Guay et al., 2019; Salsman et al., 2015).

People with dementia may rely on others to support their spiritual needs, as progressive symptoms—such as memory loss, impaired communication, and reduced mobility—can limit a person’s ability to independently access spiritual resources, participate in religious or spiritual practices, or articulate their spiritual concerns (Daly et al., 2013). Spiritual care has been defined as listening to and guiding individuals through life’s difficult questions (Creel & Tillman, 2008; Meraviglia et al., 2028). Newlin et al. (2002) and as as caring for important values in people’s lives. Other perspectives highlight that spiritual care involves connecting individuals to their sources of meaning (Jackson et al., 2022), and providing emotional support, dignity, and comfort (Camacho-Montaño et al., 2021).

For this scoping review, we define spirituality as ‘at the core and essence of who we are, that spark which permeates the entire fabric of the person and demands that we are all worthy of dignity and respect. It transcends intellectual capability, elevating that status of all humanity to that of the sacred’ (McSherry, 2009 as cited in McSherry & Smith, 2012, p. 118). Throughout this article, we use the term spirituality to encompass religion, viewing religion as a specific expression within the broader spiritual domain. This approach aligns with other research, which views spirituality as an overarching term, with religiosity being a specific form of spirituality that involves, within a faith tradition, finding purpose and meaning through connections to entities beyond oneself, such as deities (Ødbehr et al., 2017).

## Aim and Objectives

### Aim

To systematically map the research conducted on spiritual interventions developed to provide spiritual support for people living with dementia and their caregivers across the disease trajectory.

### Objectives

- To describe the characteristics and outcomes of spiritual interventions designed to provide spiritual support for individuals with dementia and their caregivers across the dementia trajectory.
- To describe in general terms the population (e.g., stage of dementia), designs and methodologies that have been used.
- To establish key findings of the evaluations and to summarise the benefits of reported spiritual interventions.

## Methods

### Design

Scoping reviews are valuable for exploring the breadth, scope, and characteristics of existing research on a particular subject. They help in summarizing and disseminating findings from a diverse and complex body of evidence (Arksey & O'Malley, 2005). The choice to undertake a scoping review instead of a systematic review was influenced by the ambiguous definition of ‘spiritual support.’ In this review spiritual support covered; interventions (both individual and group), service delivery models, programmes or toolkits to support the delivery of care, approaches to care or support and specific activities. This review protocol adhered to the reporting guidelines set by the Preferred Reporting Items for Systematic Reviews and Meta-Analyses (PRISMA) Extension for Scoping Reviews (Tricco et al., 2018). The review protocol was published on OSF (Wolverson et al., 2024).

### Search Strategy

A search was completed in December 2024 and updated in February 2025 of the following key international databases; MEDLINE (Pubmed), CINAHL, PsycINFO, EMBASE, Web of Science and The Cochrane Library. Databases were selected for their coverage of medical, nursing, psychological and interdisciplinary research. Reference list searches and forward citation searches were completed for included studies.

Search terms were developed based on other reviews within the area and in consultation with an academic librarian and covered spirituality, dementia, psychosocial and intervention domains (Supplement A, search terms).

### Inclusion Criteria

A study had to meet all inclusion criteria across population, concept, context, and study design to be included in the scoping review (Table 1). To be included, articles must have been published in the following languages: Danish, Dutch, English, French, German, Italian, Norwegian, Portuguese, Spanish or Swedish as these were the languages spoken by members of the INTERDEM palliative and end of life care taskforce. No date limits were set.

**Table 1.** Inclusion and Exclusion Criteria

Population	
Inclusion criteria	The population studied must consist of persons with dementia of any cause, or family or professional caregivers, or volunteers providing or facilitating spiritual support
Exclusion criteria	Studies focusing on/including mixed populations of dementia and non-dementia (eg, cancer), when findings are not reported by disease group
Intervention	
Inclusion criteria	Spiritual interventions (both individual and group), service delivery models, programmes or toolkits to support the delivery of care, approaches to care or support, approaches and adaptations to worship to make it inclusive, specific activities, with the root words spirit* or relig* or these root words’ derivatives within the title and abstract
	Throughout the article, religion is defined as falling conceptually under the broader term of spirituality
	Interventions do not need to have been developed specifically for people with dementia or their caregivers but must have been applied to people with dementia and /or their caregivers (with or without adaptation)
Exclusion criteria	The concept of spirituality is not reported/explicitly referred to
	Concepts of spirituality have to be reported explicitly rather than only being embedded within other constructs (e.g., psychosocial concerns)
	Spirituality-based quantitative variables are only presented as descriptors, predictors, or correlates
	Interventions that focus on completion of medical directives, pharmacological inventions, or an assessment of the participant’s psychosocial needs without an action or set of actions designed to modify an outcome or achieve changes
Setting/Context	
Inclusion criteria	Studies taking place in any care setting, (combination of) settings: community dwelling, patient’s home, outpatient/private clinic, community, day care centre, emergency department, hospital, hospital discharge towards home or nursing home, nursing home, residential care home, long-term care, memory clinics
Study Design	
Inclusion criteria	Original empirical research, papers must collect and analyse new data to answer a specific research question or test a hypothesis i.e. RCTs, observational studies, cross-sectional studies, case-control studies, case series or case reports. Any form of qualitative research (e.g., observations, interviews, focus groups), evaluation of local programme or audits)
Exclusion criteria	Editorials, letters, opinions, conceptual papers, dissertations, books or book reviews, conference proceedings, posters or abstracts, literature reviews, meta-analyses, protocols
	Papers were not included that listed ideas for spiritual support without presenting original empirical data or systematic analysis

*Indicate the words as in the form of search terms.

### Study Selection

To determine inclusion eligibility, title and abstracts from the results of the search were independently screened by two authors (SS, AFdP) using Rayyan software. Eligible studies advanced to the next stage and were read in full by two independent reviewers who were members of the review team (AB, J-BM, SV, SE, TB, RK, M-JG), who confirmed their eligibility using the predetermined criteria. A third reviewer (EW) read and agreed all the included articles. At each stage any disagreements were resolved through discussion with two further members of the review team (JvdS and KHD). One area of disagreement, resolved through a discussion with the INTERDEM taskforce on palliative and end of life care, was related to articles about Namaste Care. The decision was made not to include articles on Namaste Care, as whilst this is a holistic and person-centred psychosocial approach, it is not primarily a spiritual care intervention. Namaste does not explicitly include components specific to spiritual care, rather physical and sensory activities are primarily a means to connect with people. Dignity Therapy was also excluded from this review on the basis that it was not originally conceived as a spiritual care intervention, despite its later associations with spiritual well-being.

### Data Charting, Data Items, and Synthesis

Data were extracted by EW and organised into a Microsoft Excel spreadsheet developed by the team. Data items included title, authors, year of publication, country, participant demographics, intervention, methods, outcome measures and key findings. A narrative review of the findings was developed to address the review questions, grouping similar interventions.

### Quality Appraisal

The quality of the included studies was assessed using the mixed methods appraisal tool (MMAT; Hong et al., 2018). The MMAT provides a comprehensive set of tools designed to evaluate the validity, results, and relevance of various types of research studies, including randomized controlled trials, qualitative studies, and systematic reviews. These tools are widely recognized for their ease of use and effectiveness in ensuring a rigorous appraisal process. Each was scored “Yes,” “No,” or “Can’t tell,” and the overall quality was determined based on the number of “Yes” responses. Where studies reported both qualitative and quantitative data but did not report to be a mixed methods study (i.e. not aiming at full integration) the qualitative and quantitative components were analysed separately. Studies with higher scores are considered to have better methodological quality. Quality thresholds (≥ 70% = high quality, ≥ 50% and < 70% = moderate quality, ≤ 50% = poor quality) were imposed, based on previous studies (Feast et al., 2020). No papers were excluded based on quality score. All studies were scored independently by two members of the research team (EW, KHD, JvdS) any disagreements were resolved through discussion (Supplement B).

### Involvement of Experts by Experience

The results of this scoping review were shared with seven people living with dementia and six caregivers or former caregivers (experts by experience) through two online focus group meetings (via Microsoft Teams) and one individual email exchange. A short PowerPoint presentation was used to provide an accessible overview of the included studies and their findings. Participants were invited to provide feedback on the types and content of interventions identified in the review. During the meetings, open-ended questions guided the discussion, and participants were encouraged to share their perspectives freely. Notes were taken during the sessions with participants’ verbal consent. Separately, the email respondent was sent the slide deck and invited to provide written feedback in response to the same set of guiding questions.

The data collected were thematically summarized by two members of the research team (TB, EW), who reviewed the notes and responses to identify recurring insights, concerns, and suggestions. While these experts by experience were not involved in the initial design or decision to undertake the scoping review, their involvement at the interpretation stage added significant value. Their contributions helped highlight gaps in the literature and ensure that the reviews recommendations reflected the priorities of those directly affected by dementia.

## Results

### Study Selection and Characteristics of Included Studies

**Figure 1.**
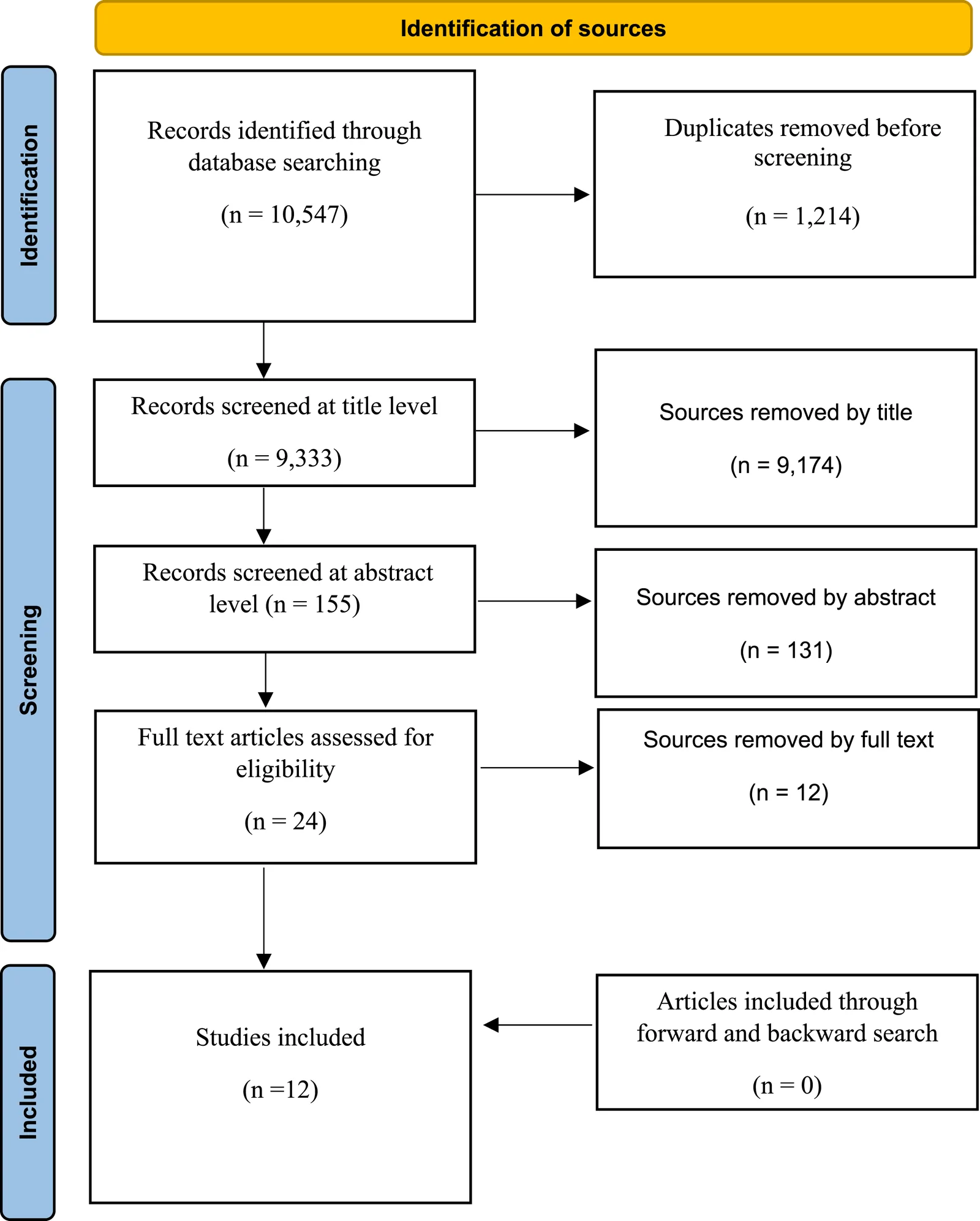
Study Selection Flow Diagram

The process of data selection is shown in Figure 1. The initial search retrieved 10,547 articles. After removing 1214 duplicates, the remaining 9333 were reviewed by title and 9178 were excluded. Then 155 were reviewed by abstract and 131 were excluded. A full review was performed on 24 articles. Among these, 12 were excluded because they did not align with the review criteria. The most common reason for exclusion was that studies described spiritual needs but did not aim to provide spiritual support. Finally, 12 articles were included in the review.

Retrieved studies were published between 2009 and 2024 and all were written in English. Studies originated from the USA (n = 4), Europe (n = 4), Australia (n = 2), Taiwan (n = 1), and Canada (n = 1). Studies were mainly concerned with people with dementia living in the community (n = 8), in residential care (n = 3) and one (n = 1) had a mixed sample from both residential and community settings.

Study populations included professionals only (church leaders, chaplains and dementia care professionals, n = 4), caregivers only (current and former, n = 2), people with dementia only (n = 5) and one study with mixed populations. Studies that involved participants with dementia (n = 6), included mainly those with mild to moderate dementia (n = 4), one (n = 1) study focused on moderate to advanced dementia and one (n = 1) had a mixed sample across different stages of dementia.

Studies included a range of designs and methods including RCT (n = 1), pre and post evaluation (n = 1), participatory research (n = 2), mixed methods (n = 3), qualitative case study (n = 1), qualitative descriptive studies (n = 3) and hermeneutic phenomenological design (n = 1).

Studies using outcome measures (n = 5) for people with dementia included measures looking at specific behaviours, hope, life satisfaction, spiritual wellbeing. For caregivers, measures of depression, burden, problem severity, positive aspects of caring, the dyadic relationship and satisfaction with counselling were used. Services studies included measures to assess the care environment and care practices. The MMAT quality scores varied considerably (Table 2).

**Table 2.** Included Studies

Author, year and country		Aim/Design	Participants (n)/stage of dementia	Setting	Intervention	Outcome measures	Key findings	MMAT
Direct Spiritual Support Interventions								
Kirkland et al. (2014) Canada		Analyse the outcomes of a co-led combined spiritual care and music therapy (SCMT) group offered to persons with moderate to advanced dementia living in an extended care setting	Residents with moderate to advanced dementia (n = 9) of a residential care	Residential care	Combined spiritual care and music group. Weekly group meeting for up to 1 hour	Dementia Care Mapping (Bradford Dementia Group, 1997) – looking at behavioural category codes	Dementia Care Mapping indicated 85% participant engagement, with Mood and Engagement (ME) scores, reflecting sustained positive mood. Interviews indicated that group participation promoted social connection, spiritual fulfilment, reciprocity, and a sense of community.	100%
		Mixed methods	Aged 52–99 years average 75 years					
			Male 1 Female 8					
MacKinlay and Trevitt (2010) Australia		Evaluate the effectiveness of spiritual reminiscence as a method for assisting people with dementia to find meaning in their life situation.	Participants with dementia (n = 113)	Residential care	Spiritual reminiscence group. Groups meetings weekly for either 6 or 24 weeks in a facilitated, spiritual reminiscence session.	N/A	Spiritual reminiscence supported meaning-making and coping; participants reported increased self-worth, identity affirmation, and a desire to continue meeting post-intervention.	40%
		Qualitative	13% male 87% female					
			Mean age 83.37					
			Average MMSE score 18.12 entry and 16.09 upon completion study					
Wu and Koo (2016) Taiwan		Investigate the effects of spiritual reminiscence on hope, life satisfaction, and spiritual wellbeing in older people in Taiwan with mild or moderate dementia	Patients with mild or moderate dementia (n = 103 participants in total, n = 53 received the intervention and n = 50 control group) recruited from a medical centre in central Taiwan.	Community	6-week spiritual reminiscence group	The Herth Hope Index (Herth, 1992), the Life Satisfaction Index (Neugarten et al., 1961), the Spirituality Index of Wellbeing (Daaleman & Frey, 2004)	The intervention significantly improved hope, life satisfaction, and spiritual well-being in older adults with mild to moderate dementia, with group-by-time interaction effects showing greater gains compared to the control group	80%
		RCT	Male: 31% Female: 69%					
			Mean age 73.6 years					
			MMSE score mean 23, 90% mild dementia and 10% moderate dementia					
Interventions for caregivers								
Glueckauf et al. (2009) United States.		Initial evaluation of a program for training faith community nurses to conduct cognitive–behavioural and spiritual counselling for rural dwelling dementia caregivers.	Spousal caregivers (n = 2) of a person with dementia	Community	Spiritual behavioural counselling for carers. 12 one-hour sessions delivered biweekly in caregivers own homes over a period of 6 months.	Counselling comfort scale (Glueckauf et al., 2007a), Counselling efficacy scale (Glueckauf et al., 2007b), Counselling workshop satisfaction survey (Glueckauf et al., 2007c), Glueckauf’s Issue Severity Scale (Glueckauf, 2000), Centre for epidemiological studies depression scale (Radloff, 1977)	CBT and spiritual counselling for rural dementia caregivers led to reduced depression and improved problem-solving and faith community nurses reported increased confidence and efficacy post-training.	40%
		Pre-post evaluation	50% male 50% female					
			Age range 71–76, mean 73.5 years.					
Toolkits to support/upskill professionals								
Haufe, Leget, et al. (2024) Netherlands	To develop the Diamond conversation model for early-stage dementia to enable professionals to provide better spiritual support.		Chaplains (n = 4), psychologists (n = 3) and case managers (n = 4)	Community	A structured framework designed to guide professionals in providing conversational support for discussing individuals’ spiritual needs	N/A	Participatory development of the Diamond Conversation Model identified key spiritual tensions (e.g., self-worth vs. loss, attachment vs. letting go); model offers a framework for supporting existential and spiritual needs in early-stage dementia.	100%
	Participatory research		Age 46–63 mean 52.73 years					
			Male 17% Female 83%					
Haufe, Teunissen, and Leget (2024) Netherlands	To pilot test support approaches that may enable professionals to better conduct conversations with attention for existential and spiritual issues		Case managers (n = 5) and psychologist (n = 1) Age range professionals 34–56, mean age 42.6 years	Community	A structured framework for professionals to provide conversational support for discussing individuals’ spiritual needs	N/A	Pilot study using the Diamond model identified five conversational approaches to support existential and spiritual needs; professionals reported increased confidence, and clients experienced enhanced meaning, self-worth, and emotional connection	100%
	Participatory research		Who spoke to people with mild to moderate dementia (n = 62) and caregivers (n = 36)					
			Those with dementia 71% female 29% male.					
			Age range of those with dementia 54–96 years.					
Mumby (2023) UK	Establish methods to assess spiritual health and provide support for those with language impairments		One person with a diagnosis of logopenic progressive aphasia	Community	A toolkit for assessing and supporting spiritual health for mild to moderate dementia.	N/A	Use of the WELLHEAD Toolkit enabled a person with logopenic aphasia to explore spiritual concerns and identity through storytelling; supported meaning-making, emotional expression, and connection	100%
	Qualitative		Male					
			Age 65 years					
A caring and living environment to support spirituality								
Burke et al. (2018) Australia		To describe how an Australian age care provider, has embraced the National Guidelines for Spiritual Care in Aged Care for people with dementia	Residential care managers (n = 18), nurses (n = 24), care assistants (n = 55), lifestyle staff (n = 26), chaplains (n = 13) all working across 19 care homes	Residential care	Service evaluation conducted using National guidelines for spiritual care in aged care settings	Person-Centred Environment and Care Assessment Tool (PCECAT, Burke et al., 2016)	Staff surveys showed high recognition of spiritual care importance (90%) but low confidence (30%); interviews highlighted barriers (e.g., time, training) and valued person-centred approaches like life story work and rituals	Qual 80%, quant 100%
		*Mixed methods						
Toivonen et al. (2023) Finland		To further understanding about the spirituality-supportive elements of a caring and living environment from the perspective of older people with dementia and their family members	People with dementia (n = 10) and caregivers (n = 9)	Community and Residential care	Developing spiritually supportive physical Environments in residential care.	N/A	A caring environment that supports spirituality in dementia was characterised by safety, dignity, meaningful relationships, and connection to nature; staff presence and attentiveness were essential to fostering spiritual well-being	100%
		Qualitative	Different stages of dementia					
			People with dementia Male 3 Female 7 Age range: 76–97 years					
			Caregivers Male 1 Female 8 Age range: 45–80					
Culturally inclusive worship								
Epps et al. (2020) United States		To explore how to design or modify worship services to support African-Americans living with dementia	Church leaders (n = 8), family member to a person with dementia (n = 1), current and former caregivers (n = 5), community members and service providers (n = 4) with some individuals having overlapping roles.	Community	Exploring ways of adapting worship services to enhance accessibility, engagement, and inclusivity	N/A	Interviews with caregivers, church leaders, and service providers identified four key elements for dementia-friendly worship: simplicity, support, imagery and sound, and music**.** Support for caregivers also needs to be provided. Operating separate services might exacerbate stigma.	100%
		Qualitative	Male 17% Female 83%					
			Ages ranged from 38 to 71 years, mean age 56.5 years					
Epps et al. (2021) United States		To explore ways African American churches can be dementia-friendly to support families affected by dementia	Church leaders (n = 8), family member to a person with dementia (n = 1), current and former caregivers (n = 5), community members and service providers (n = 4) with some individuals having overlapping roles.	Community	Exploring how churches can be more dementia-friendly	N/A	African American church leaders and members identified strategies for creating dementia-friendly churches, including education, caregiver support, inclusive worship, and fostering a culture of acceptance to better support families affected by dementia.	100%
		Qualitative	Male 17% Female 83%					
			Ages ranged from 38 to 71 years, mean age 56.5 years					
Sainz et al. (2023) United states		To determine the feasibility and preliminary efficacy of culturally appropriate online worship services for Black dementia caregivers	Christian African American family caregivers (n = 24)	Community	Culturally appropriated tailored online worship services	Zarit Burden Interview (Zarit et al., 1980), Perceived Stress Scale (Cohen et al., 1983), Positive Appraisal of Care (Yamamoto-Mitani et al., 2021), Dyadic Relationship Strain Scale (Sebern & Whitlatch, 2007)	Tailored online worship services led to significant reductions in stress and improvements in spiritual well-being (quantitative); qualitatively, caregivers described emotional relief, spiritual renewal, and a sense of stillness, connection, and cultural affirmation.	100%
		Mixed methods	Male 4% Female 96%					
			Age range 45–55 years					

*Study does not state design therefore quantitative and qualitative approaches components were evaluated separately.

### Spiritual Support Interventions

Table 2 provides an overview of the included studies. Interventions are categorised in the narrative below which provides details on key features.

#### Direct Spiritual Support for People With Dementia

Three studies reported the results of interventions designed to provide spiritual support directly to individuals with dementia. All three studies reported group interventions, two (Kirkland et al., 2014; MacKinlay & Trevitt, 2010) were conducted in residential care and one (Wu & Koo, 2016) in the community.

Kirkland et al. (2014) evaluated a co-led combined spiritual care and music therapy group (SCMT) for people with moderate to advanced dementia living in residential care. The researchers were interested in the meaning and significance of the group for participants’ self-expression and wellbeing. The weekly group included 10–14 participants for one-hour sessions. Sessions included familiar songs and rituals using prayers, poems and images and finishing with individual blessings. Each session was guided by a theme and began and ended in the same way but was also designed to be flexible and responsive to participants’ mood and input.

The group was evaluated using a mixed methods approach incorporating both interviews and dementia care mapping (DCM) (Bradford Dementia Group, 1997), an observational tool used in care settings to assess and improve the quality of care for people with dementia by focusing on their emotional, social, and physical wellbeing. Nine participants were interviewed all of whom had participated in the group for three months. DCM was completed for seven sessions providing information about the psychosocial context of the participants.

DCM indicated high levels of engagement and no evidence of ill-being during sessions. High engagement and mood scores were particularly noted by the authors when participants were connected with music, such as singing or tapping their feet. The DCM tool, however, may not have fully captured all positive experiences, such as the quiet, reflective engagement of some participants. Further, for people who are distressed, little engagement might be a positive outcome, it might suggest a person is relaxed and calm.

Interviews indicated that participants experienced enjoyment and comfort from listening to music, and described the group as providing social connection, a sense of belonging, community, and mutual support. These themes were consistently mentioned across interviews. MacKinlay and Trevitt’s (2010) group intervention focused on spiritual reminiscence, defined as a way of telling one’s life story with a focus on meaning and purpose. The study involved 113 people with dementia living in residential care facilities, who participated in small weekly spiritual reminiscence groups over periods of either six weeks or six months. Group sizes were adjusted based on the participants’ level of dementia and communication abilities, with smaller groups for those with more advanced dementia. Sessions lasted between 30 min to 1 hour.

Qualitative data was gathered from session transcripts, individual interviews, observer journals, and small group discussions. Participants in the 24-week groups expressed a desire to continue meeting after the research project ended, perhaps demonstrating that a limited number of therapy sessions would miss out on a wish for a continued connection. The researchers found that the group sessions helped individuals relate to each other differently than in the general environment of the residential care facility by providing participants opportunity to discuss important relationships and connect with others. It helped them find meaning in their current lives and develop strategies to cope with the changes of later life, including the loss of significant relationships and increasing disability. The sessions allowed people to express their fears, hopes, and future expectations as they approached the end of their lives. The authors state that quantitative data was also gathered and will be reported elsewhere but our literature search did not locate this.

The shared conclusions from both of these studies (Kirkland et al., 2014; MacKinlay & Trevitt, 2010) in residential care emphasize the importance of keeping group sizes small (no more than six people) to create a supportive and intimate environment. They also highlight the crucial role of skilled facilitators in engaging participants and adapting to their diverse needs. Additionally, both studies note the challenges of delivering such programs in busy settings and stress the need for flexible programs that allow facilitators to respond to the unique characteristics of each group member.

Wu and Koo (2016) also examined a spiritual reminiscence intervention, focusing on people with mild to moderate dementia living in the community. This RCT examined the effects of a six-week spiritual reminiscence intervention on hope, life satisfaction, and spiritual wellbeing in older people in Japan. Participants included 103 people with dementia recruited from a medical centre, who were randomly assigned to either the 6-week spiritual reminiscence group (n = 53) or the control group who just completed the measures. The intervention consisted of 1-h sessions with groups of three to six participants, involving for example, singing, storytelling and arts-based activities. The sessions focused on six themes: meaning in life, relationships and connections, hope fears and worries, growing older and transcendence, spiritual and religious beliefs and spiritual and religious practices.

The Herth Hope Index (Herth, 1992), Life Satisfaction Index (Neugarten et al., 1961) the Spirituality Index of Wellbeing (Daaleman & Frey, 2004) and the Mini Mental State Examination (Folstein et al., 1975) were administered before and after the intervention. All measures showed an increase in the intervention group and a decrease in the control group. The interaction terms between group and time for the four scores were significant (*p* < 0.001), indicating the changes over time in these scores were significantly different between the two groups.

#### Interventions for Caregivers

We found one study that explored integrating spirituality into a psychological intervention for family caregivers. Glueckauf et al. (2009) evaluated a programme of training for faith community nurses to conduct cognitive-behavioural and spiritual counselling (CBSC) for caregivers of people with dementia living in rural areas. They present two case studies with pre and post evaluation on the use of CBSC for treating depression. The authors report that preliminary outcomes were promising suggesting that training faith community nurses to deliver this combined approach was effective. Caregivers who received the counselling self-reported significant improvements in their depression and felt better supported in their caregiving roles. The paper highlights the potential benefits of integrating mental health and spiritual support for caregivers in a rural setting. However, more extensive research is needed to explore the long-term effectiveness of the approach. Caregiver’s ambivalence in thinking about end of life and the ‘possibility of relapse’ given the chronic and progressive nature of dementia were two major challenges that nurses faced. The study highlighted the importance of supervising faith nurses —registered nurses who integrate spiritual care into their practice, often working within faith-based communities or organizations— when delivering interventions, providing them with a space to reflect on their own views on spirituality, and ensuring they have a connection to health services for escalating concerns.

#### Toolkits to Support /Upskill Professionals

Three papers examined tools designed to help professionals discuss and assess the spiritual needs of individuals with dementia, and to provide appropriate support.

The Diamond conversation model (Haufe, Leget, et al., 2024) focuses on addressing key existential and spiritual issues. This model, originally used in palliative care, was adapted for use in dementia through participatory research involving chaplains, psychologists, and case managers.

The model identifies five central polarities thought to reflect the existential and spiritual tensions experienced by individuals with dementia. These polarities include balancing the need to maintain a sense of self with feeling valued by others, finding direction in life while adapting to changes in abilities, managing the need for attachment while letting go of past ways of relating, navigating the intensity of long-term memories alongside the decline of short-term memory, and surrendering to one’s life situation while seeking certainty and meaning. The Diamond model provides questions and prompts to facilitate conversations around these polarities, to help professionals support the spiritual and existential needs of individuals with early-stage dementia. In their second study, Haufe, Teunissen, and Leget (2024) then set to refine and test the model. This second study involved six dementia care professionals working with 62 people with dementia and 36 family members, focusing on those with mild to moderate dementia. Using participatory action research, the study included two cycles: analysing current support and developing strategies, then testing these strategies, reflecting on their effectiveness, and creating new approaches.

Findings showed that professionals need help recognizing existential or spiritual issues in conversations. To address these issues, professionals must pause their own agenda and explore tensions, which often indicate underlying existential or spiritual concerns.

Reflecting on the development and application of the Diamond conversation model, the authors note several limitations that should be acknowledged. First, the research did not directly involve people with dementia and their families in discussions about the specific issues the model addresses. This lack of direct input may limit the model’s comprehensiveness and relevance to the lived experiences of those it aims to support. Additionally, the researchers’ Christian background may have influenced the model’s development, potentially introducing a bias towards Christian perspectives on spirituality. This could affect the model’s applicability and acceptance among individuals from diverse religious or spiritual backgrounds. A further limitation of the study is the lack of direct feedback from people with dementia and their families and the researchers highlight the need to consider a broader range of spiritual perspectives to enhance the model’s inclusivity and effectiveness.

Mumby (2023) presents a single case study on the use of the WELLHEAD Toolkit for assessing and supporting spiritual health. Co-created with people with aphasia, the toolkit provides structured resources and communication support for discussing meaning and purpose in life. It covers four dimensions: connecting, becoming, transcendence, and personal value, and includes starter questions, key words, and picture prompts. Goal setting is also part of the interview process.

In the case study, the participant, diagnosed with logopenic progressive aphasia (LPA), engaged in a 60-min interview with a 10-min break. The interview was recorded and analysed using thematic analysis. The case study indicates that the toolkit positively impacted the participant’s wellbeing, enabling the person to articulate their spiritual needs and goals despite their aphasia. However, since this is a single case, further research is necessary to determine the toolkits applicability to a broader population.

#### A Caring and Living Environment to Support Spirituality

The role of the environment to support spiritual wellbeing was explored in two studies.

Toivonen et al. (2023) explored spirituality-supportive elements of environment from the perspective of ten older people with dementia and their caregivers. Participants lived at home or in a long-term care facility, were at different stages of dementia, but all had capacity to consent to the research. Using photo elicitation and dyadic interviews, the results demonstrated how the living environment can help people with dementia connect to what is important in their lives, thus supporting their spirituality. Practical elements included spiritual literature, parish activities, spiritual radio, television or music, praying, art, nature, meaningful relationships, and nurses respecting the person’s religion. Environments that hold meaningful memories make people feel safe, comfortable, and supported. They also foster a sense of belonging to others and the local area, creating meaning and purpose in life. Additionally, environments can facilitate connections with the wider world and nature, which are important aspects of spirituality. Despite being a small study and in one specific culture (Southern Finland), it portrays significant practical implications for supporting the spiritual wellbeing of people with dementia in new environments.

Extending the environment to also consider the care environment, Burke et al. (2018) examined how the National Guidelines for Spiritual Care in Aged Care (Meaningful Ageing Australia, 2016) were implemented for people with dementia in Australia. These guidelines emphasize four principles: a whole-organization approach, relational care, the universal responsibility of spiritual care, and fostering growth and flourishing.

The study was a 12-month quality improvement project in 18 residential homes, utilizing interviews, observations, and policy reviews. The researchers used their Person-Centred Environment and Care Assessment Tool (PCECAT) (Burke et al., 2016) to evaluate care and environmental aspects, ensuring alignment with Australian residential care standards. The PCECAT comprised 76 items across three domains (organizational culture, care and activities, and physical layout and design), to identify improvement areas and ensure person-centred care.

The assessment showed high adherence to the guidelines’ principles. Spiritual support witnessed included chaplaincy and pastoral care embedded as key services, with devotions held in chapels or private prayer rooms. Residents received visits during hospital stays, engaged in spiritual reminiscence, and enjoyed one-on-one time in their rooms with the chaplain. Activities like reading poetry, playing favourite music, and spending time gardens were meaningful, while holding religious objects and listening to quiet music fulfilled significant rituals. Church group representatives visited upon request, and end-of-life wishes, such as a beach visit for a resident, were honoured. Chaplains assessed spiritual needs with residents and families, recorded preferences, and provided comfort during palliative care, fostering trust and emotional support. The authors concluded that a whole-system approach, integrating spirituality within organizational values and services, is essential to meet individual spiritual needs.

#### Culturally Inclusive Worship

Three studies explored how to provide culturally appropriate worship for the Christian African American community affected by dementia.

Epps et al. (2020) conducted interviews with church leaders and caregivers (n = 12) to explore how worship services can be designed or modified to be more inclusive of those with dementia and their caregivers. They identified four key components for creating dementia-friendly worship services. First, simplicity was seen as crucial, involving shorter sessions, simpler hymns, and straightforward sermons. Second, the use of imagery and sound to enhance engagement. Third, providing support through additional staff and offering emotional care for caregivers was seen as essential. Finally, music was highlighted as a powerful tool to promote engagement, elevate mood, and stimulate memory.

Epps et al. also explored how churches could become more dementia friendly (2021). A welcoming environment for the person and the caregiver was seen as key, started by being greeted warmly at the door. To promote inclusion and comfort, participants described the importance of advertising the church as dementia friendly and ensure the building is accessible for this population (considering lighting, parking, signage, etc.). In order for the church congregation to be understanding, accepting and non-judgemental, it was proposed that education about dementia was needed. Finally, it was proposed that churches need to value their members’ wellbeing, and this was seen to include providing support for caregivers including home visits and providing respite.

Based on this learning, Sainz et al. (2023) explored the feasibility and effectiveness of culturally appropriate online worship services for Christian Black caregivers of people with dementia. The intervention consisted of a six-week course of 15-min videos of tailored worship services designed for caregivers to watch with their relatives with dementia. Caregivers’ perceptions of stress (Perceived Stress Scale, Cohen et al., 1983), burden (Zarit Burden Interview, Zarit et al., 1980), positive aspects of caring (Positive Appraisal of Caregiving, Yamamoto-Mitani et al., 2021), and relationships (Dyadic Relationship Strain Scale, Sebern & Whitlatch, 2007) were assessed using self-report measures before and after the intervention. Observations of dyads during the intervention and post-intervention interviews with caregivers were also conducted. Worship attendance was recorded.

Twenty-four caregivers, mostly female adult children, participated in the study. Twenty-one dyads watched all six video sessions, and 15 caregivers completed pre- and post-intervention measures, which showed no significant differences. However, interviews and observations highlighted the value of shorter sessions for engagement and the flexibility of the online format. Caregivers reported that culturally appropriate (with songs and hymns that are easily recognisable) shared worship uplifted their spirits and provided moments of closeness with their relatives.

The authors conclude that religiosity plays a significant role in the lives of Black family caregivers and that these services can provide a sense of familiarity, understanding, and community that is particularly meaningful, helping caregivers feel more supported and connected.

## Discussion

This review described and categorised interventions developed to provide spiritual support across the dementia trajectory. Interventions have been developed for people with dementia, for caregivers, to improve staff skills and care environments and to adapt worship to make it more inclusive.

Interventions developed for people with dementia and caregivers, involved the addition of spiritual support to existing psychosocial interventions, including music groups, cognitive behavioural therapy (CBT) and reminiscence. These interventions centred on helping people identify a sense of meaning and purpose in life.

It is notable that interventions developed for people with dementia were all group-based. This likely reflects the relational aspects of spirituality and the importance of connectedness as key components of spirituality (Dyson et al., 1997). Group interventions, particularly in residential settings may be considered more cost-effective, given the limited resources for one-on-one interventions. However, what was notable was that interventions highlighted the importance of flexibility and skilled facilitators to address the unique needs of each participant. This flexibility was demonstrated through shorter sessions and looser agendas, allowing facilitators to adapt to the varying needs and preferences of participants. Moreover, the value of creative and multi-sensory activities (music, prayer, art, time outdoors) in supporting engagement was evident across spiritual interventions for people with dementia. Music was highlighted as having a special importance in five of the 12 studies (Burke et al., 2018; Epps et al., 2020; Kirkland et al., 2014; Sainz et al., 2023; Toivonen et al., 2023).

We found only one study (Glueckauf et al., 2009) exploring spiritual support for caregivers, which is surprising given the well-established value of spiritual support in enhancing well-being and improving the quality-of-care delivery in palliative care (Uzun et al., 2024). Indeed, within dementia research, spirituality and religious beliefs often emerge as critical coping mechanisms, providing strength and meaning amidst caregiving challenges (Monteiro et al., 2018).

Toolkits designed to support professionals in obtaining a spiritual history and conducting a spiritual assessment are a crucial first step in providing appropriate spiritual support. While several instruments exist (e.g., FICA (Puchalski et al., 2018)), their adaptation to the context of dementia and specific challenges such as aphasia is particularly welcome. Indeed, Britt et al. (2023) called for more dementia-specific tools such as the ones found in this review. Future research should focus on tools to support both verbal and nonverbal expressions of meaning and preferences in a person with dementia.

Creating a living environment that supports and nurtures spirituality is essential for allowing individuals to express their beliefs, values, and the things that are important to them. The creation of an appropriate physical environment for the provision of spiritual care has been conceptualised as a central attribute of spiritual care interventions in adult nursing (Ghorbani et al., 2021). For individuals with dementia, further research into spiritual nursing environments is needed across settings such as hospitals, mental health wards and day services.

Research on culturally inclusive worship is an important reminder that people with dementia and their caregivers are not a homogenous group. This review highlights that cultural differences in spiritual beliefs and practices can impact the applicability of interventions across different populations, necessitating culturally sensitive approaches to ensure inclusivity and effectiveness. As research in this area develops, it is important to consider those who require tailored support. For example, individuals with young onset dementia may struggle more with self-identity, self-perception, anger, and mental health issues following diagnosis (O’Malley et al., 2021).

The quality of evidence relating to spiritual support is varied. Sample sizes were often small and evaluation cross sectional in nature, making it difficult to know if interventions had benefits experienced over time. The diversity of protocols and outcomes associated with a lack of standardisation of interventions point to the need for further studies evaluating spiritual support in dementia care. Many of the studies were exploratory in nature and measured engagement, seeking people’s experiences and views of the intervention to assess its feasibility and acceptability. The studies that did focus on improving spiritual wellbeing and clinical symptoms such as depression suggested some benefits, but more robust trials of interventions are needed. Spiritual experiences and outcomes are highly subjective, which can complicate the measurement and evaluation of intervention effectiveness. However, these personal experiences are valuable and should be considered in the evaluation process to ensure interventions are meaningful and impactful (Jaman-Mewes et al., 2024). The involvement of people with dementia and caregivers as experts by experience in designing interventions and in shaping the future focus of research in this area is vital.

### Insights From People Living With Dementia and Caregivers

People with dementia and their caregivers strongly believe that spiritual support should be multifaceted and personalised. Spirituality means different things for different people. Information about available spiritual support should be accessible to both those diagnosed and their caregivers. They emphasised the importance of creative activities such as art, music, and time in nature for meaningful engagement and emotional relief. Counselling and reminiscence activities are valued, though the latter should be approached carefully to avoid negative experiences. It is crucial to advertise the specific aspects of spiritual sessions to ensure participants know what to expect, avoiding the imposition of potentially unwanted practices like prayer during music therapy. Support should be tailored to each individual’s unique experience, with dignified and appropriate activities that empower individuals to maintain their spirituality. The people we spoke to felt that spiritual support should not impose religious beliefs unless clearly advertised as such, in order to respect diverse backgrounds. Future research should focus on tailored spiritual support for both people living with dementia and family caregivers, and the needs of care staff in providing spiritual care. As part of this, research supporting people living with dementia should focus on the role of art, counselling, and planning for the future, meeting aspirations, and achieving goals in supporting spirituality.

## Limitations

This scoping review focused on peer-reviewed articles to examine the evidence base for, and type of spiritual support interventions. We are aware that there are resources and guides created for churches in how to provide spiritual care and support to people with dementia (e.g. Collicutt, 2017; MacKinlay & Trevitt, 2006) which were beyond the scope of this review. Although we were able to include articles written in multiple languages, all those retrieved were published in English, perhaps reflecting the language of our search terms. Additionally, we did not include the ATLA Religion Database in our search strategy, which may have limited the identification of literature specifically focused on spiritual support. Due to the high volume of search results, we also employed title-only screening in the initial phase, which may have resulted in missing relevant studies that would have been identified through abstract or full-text review.

## Conclusions

The diverse range of spiritual interventions and settings, including community, care homes, and churches, underscores that spiritual support for people with dementia is a collective responsibility. This review found a limited number of spiritual support interventions, but it is noteworthy that these were diverse and provided support throughout the entire dementia care pathway. People with dementia and caregivers felt that spiritual support should be personalised and multifaceted, incorporating creative activities and interventions tailored to the individual. The quality of evidence is varied, highlighting the need for more robust and standardized research. Involving people with dementia and caregivers in designing and evaluating spiritual interventions is crucial, as is tailored support for diverse groups and culturally sensitive approaches to ensure inclusivity and effectiveness.

## Supplemental Material


Supplemental material - Spiritual Support for People Affected by Dementia: A Scoping Review
Supplemental material for Spiritual Support for People Affected by Dementia: A Scoping Review by Emma Wolverson, Tamara Backhouse, Alice Burnand, Siren Eriksen, Andrea Fonseca de Paiva, Karen Harrison Dening, Jean-Bernard Mabire, Serena Sabatini, Jenny T. van der Steen On behalf of The INTERDEM Taskforce on Palliative and End of Life Care in Dementia

## Data Availability

The data supporting the findings of this scoping review are available within the article and its supplementary materials. Additional data may be available from the corresponding author upon reasonable request.*

## References

[bibr60-14713012251377812] Agli O. Bailly N. Ferrand C. (2015). Spirituality and religion in older adults with dementia: A systematic review. International psychogeriatrics, 27(5), 715–725. 25155440 10.1017/S1041610214001665

[bibr59-14713012251377812] Alzheimer Europe. (2021). Dementia in Europe Yearbook 2021: Dementia-inclusive Communities and Initiatives across Europe. Luxembourg: Alzheimer Europe. Retrieved from https://www.alzheimer-europe.org/sites/default/files/2022-03/Alzheimer20Europe20Dementia20in20Europe20Yearbook20202120-20Dementia-inclusive20Communities20and20Initiatives20across20Europe_0.pdf

[bibr1-14713012251377812] Arksey H. O'Malley L. (2005). Scoping studies: Towards a methodological framework. International Journal of Social Research Methodology, 8(1), 19–32. 10.1080/1364557032000119616

[bibr2-14713012251377812] Bradford Dementia Group . (1997). Evaluating dementia care: The DCM method. DCM.

[bibr3-14713012251377812] Britt K. C. Boateng A. C. Zhao H. Ezeokonkwo F. C. Federwitz C. Epps F. (2023). Spiritual needs of older adults living with dementia: An integrative review. Healthcare, 11(9), 1319. 10.3390/healthcare11091319 37174861 PMC10178032

[bibr4-14713012251377812] Burke C. Stein-Parbury J. Luscombe G. Chenoweth L. (2016). Development and testing of the person-centered environment and care assessment tool (PCECAT). Clinical Gerontologist, 39(4), 282–306. 10.1080/07317115.2016.1172532

[bibr5-14713012251377812] Burke C. Wight T. Chenoweth L. (2018). Supporting the spiritual needs of people with dementia in residential aged care. Journal of Religion, Spirituality & Aging, 30(3), 234–250. 10.1080/15528030.2018.1434852

[bibr6-14713012251377812] Caldeira S. Carvalho E. C. Vieira M. (2013). Spiritual distress—Proposing a new definition and defining characteristics. International Journal of Nursing Knowledge, 24(2), 77–84. 10.1111/j.2047-3095.2013.01234 23465219

[bibr7-14713012251377812] Camacho-Montaño L. R. Pérez-Corrales J. Pérez-de-Heredia-Torres M. Martin-Pérez A. M. Güeita-Rodríguez J. Velarde-García J. F. Palacios-Ceña D. (2021). Spiritual care in advanced dementia from the perspective of health providers: A qualitative systematic review. Occupational Therapy International, 2021(3), 1–11. 10.1155/2021/9998480 PMC8635933 34908917

[bibr8-14713012251377812] Cohen S. Kamarck T. Mermelstein R. (1983). A global measure of perceived stress. Journal of Health and Social Behavior, 24(4), 385–396. 10.2307/2136404 6668417

[bibr9-14713012251377812] Collicutt J. (2017). Thinking of you: A resource for the spiritual care of people with dementia. BRF.

[bibr10-14713012251377812] Creel E. Tillman K. (2008). The meaning of spirituality among nonreligious persons with chronic illness. Holistic Nursing Practice, 22(6), 303–309. 10.1097/01.HNP.0000339340.96005 18981809

[bibr11-14713012251377812] Daaleman T. P. Frey B. B. (2004). The spirituality index of wellbeing: A new instrument for health-related quality-of-life research. The Annals of Family Medicine, 2(5), 499–503. 10.1370/afm.89 15506588 PMC1466734

[bibr12-14713012251377812] Daly L. Fahey-McCarthy E. Timmins F. (2019). The experience of spirituality from the perspective of people living with dementia: A systematic review and meta-synthesis. Dementia, 18(2), 448–470. 10.1177/1471301216680425 27941158

[bibr13-14713012251377812] Daly L. McCarron M. Higgins A. McCallion P. (2013). ‘Sustaining Place’–A grounded theory of how informal caregivers of people with dementia manage alterations to relationships within their social worlds. Journal of Clinical Nursing, 22(3–4), 501–512. 10.1111/jocn.12003 23170780

[bibr14-14713012251377812] Delgado-Guay M. Chisholm G. Williams J. Frisbee-Hume S. Ferguson A. Spence A. Palmer J. L. Bruera E. (2019). Unmet spiritual needs impact emotional and spiritual well-being in advanced cancer patients. Supportive Care in Cancer, 27(8), 2933–2939. 10.1016/j.jpainsymman.2019.01.00630564936

[bibr15-14713012251377812] Dyson J. Cobb M. Forman D. (1997). The meaning of spirituality: A literature review. Journal of Advanced Nursing, 26(6), 1183–1188. 10.1111/j.1365-2648.1997.tb00811.x 9429969

[bibr16-14713012251377812] Epps F. Choe J. Alexander K. Brewster G. (2020). Designing worship services to support African-American persons living with dementia. Journal of Religion and Health, 59(4), 2163–2176. 10.1007/s10943-020-00993 32020382 PMC11194184

[bibr17-14713012251377812] Epps F. Heidbreder V. Alexander K. Tomlinson A. Freeman V. Williams N. (2021). A dementia-friendly church: How can the African American church support families affected by dementia? Dementia, 20(2), 556–569. 10.1177/1471301219900416 31958978 PMC11203389

[bibr18-14713012251377812] Feast A. R. White N. Candy B. Kupeli N. Sampson E. L. (2020). The effectiveness of interventions to improve the care and management of people with dementia in general hospitals: A systematic review. International Journal of Geriatric Psychiatry, 35(5), 463–488. 10.1002/gps.5280 32011033

[bibr19-14713012251377812] Folstein M. F. Folstein S. E. McHugh P. R. (1975). “Mini-mental state”: A practical method for grading the cognitive state of patients for the clinician. Journal of Psychiatric Research, 12(3), 189–198. 10.1016/0022-3956(75)90026-6 1202204

[bibr62-14713012251377812] Ghorbani M. Mohammadi E. Aghabozorgi R. Ramezani M. (2021). Spiritual care interventions in nursing: an integrative literature review. Supportive care in Cancer, 29(3), 1165–1181. 32929533 10.1007/s00520-020-05747-9

[bibr20-14713012251377812] Glueckauf R. L. (2000). The family and disability assessment system. In Touliatos J. Perlmutter B. F. Holden G. W. (Eds.), Handbook of family measurement techniques (Vol. 2, pp. 232–233). Sage.

[bibr21-14713012251377812] Glueckauf R. L. Davis W. S. Allen K. Chipi P. Schettini G. Tegen L. Jian X. Gustafson D. J. Maze J. Mosser B. Prescott S. Robinson F. Short C. Tickel S. VanMatre J. DiGeronimo T. Ramirez C. (2009). Integrative cognitive–behavioral and spiritual counseling for rural dementia caregivers with depression. Rehabilitation Psychology, 54(4), 449–461. 10.1037/a0017855 19929127

[bibr22-14713012251377812] Glueckauf R. L. Gustafson D. J. Davis W. S. Byrd V. (2007a). Faith community nurse counseling comfort scale. Florida State University, Department of Medical Humanities & Social Sciences, College of Medicine.

[bibr23-14713012251377812] Glueckauf R. L. Gustafson D. J. Davis W. S. Byrd V. (2007b). Faith community nurse perceived counseling efficacy scale. Florida State University, Department of Medical Humanities & Social Sciences, College of Medicine.

[bibr24-14713012251377812] Glueckauf R. L. Gustafson D. J. Davis W. S. Byrd V. (2007c). Faith community nurse workshop satisfaction survey. Florida State University, Department of Medical Humanities & Social Sciences, College of Medicine.

[bibr25-14713012251377812] Haufe M. Leget C. Potma M. Teunissen S. (2024). Better spiritual support for people living with early stage dementia: Developing the diamond conversation model. Dementia, 23(1), 91–108. 10.1177/14713012231213907 37934923 PMC10797830

[bibr26-14713012251377812] Haufe M. Teunissen S. Leget C. (2024). How to provide existential and spiritual support to people with mild to moderate dementia and their loved ones. A pilot study. PLoS One, 19(3), Article e0298783. 10.1371/journal.pone.0298783 38536786 PMC10971586

[bibr27-14713012251377812] Herth K. (1992). Abbreviated instrument to measure hope: Development and psychometric evaluation. Journal of Advanced Nursing, 17(10), 1251–1259. 10.1111/j.1365-2648.1992.tb01843 1430629

[bibr28-14713012251377812] Hong Q. N. Pluye P. Fàbregues S. Bartlett G. Boardman F. Cargo M. Dagenais P. Gagnon M. P. Griffiths F. Nicolau B. O’Cathain A. (2018). Mixed methods appraisal tool (MMAT), version 2018. Registration of Copyright, 1148552(10), 1–7. 10.1016/j.jclinepi.2019.03.008 30905698

[bibr29-14713012251377812] Hui E. K. Tischler V. Wong G. H. Y. Lau W. Y. T. Spector A. (2021). Systematic review of the current psychosocial interventions for people with moderate to severe dementia. International Journal of Geriatric Psychiatry, 36(9), 1313–1329. 10.1002/gps.5554 34350626

[bibr30-14713012251377812] Jackson D. Erwich R. Flynn E. Olorunnisola T. S. (2022). Equipping families and friends to offer spiritual care to people living with dementia: Findings from a meta-synthesis. Religions, 13(5), 462. 10.3390/rel13050462

[bibr31-14713012251377812] Jaman-Mewes P. de Oliveira M. C. D. S. Mazotti M. R. de Goés Salvetti M. (2024). Spiritual care interventions for palliative care patients: A scoping review. Palliative & Supportive Care, 22(5), 1449–1468. 10.1017/S1478951524000592 39136153

[bibr32-14713012251377812] Kirkland K. Fortuna M. C. Kelson E. Phinney A. (2014). Music therapy and spiritual care for persons with dementia: A mixed-methods study. Canadian Journal of Music Therapy, 20(1), 10–37.

[bibr33-14713012251377812] MacKinlay E. Trevitt C. (2006). Facilitating spiritual reminiscence for older people with dementia: A learning package. Centre for Ageing and Pastoral Studies.

[bibr34-14713012251377812] MacKinlay E. Trevitt C. (2010). Living in aged care: Using spiritual reminiscence to enhance meaning in life for those with dementia. International Journal of Mental Health Nursing, 19(6), 394–401. 10.1111/j.1447-0349.2010.00684 21054725

[bibr35-14713012251377812] McDermott O. Charlesworth G. Hogervorst E. Stoner C. Moniz-Cook E. Spector A. Csipke E. Orrell M. (2019). Psychosocial interventions for people with dementia: A synthesis of systematic reviews. Aging & Mental Health, 23(4), 393–403. 10.1080/13607863.2017.142303 29338323

[bibr61-14713012251377812] McSherry W. Smith J. (2012). Spiritual care. In McSherry W. McSherry R. Watson R. (Eds.), Care in nursing: Principles, values and skills (pp. 117–134). Oxford University Press.

[bibr36-14713012251377812] Meaningful Ageing Australia . (2016). National guidelines for spiritual care in aged care.

[bibr37-14713012251377812] Meraviglia M. Sutter R. Gaskamp C. D. Adams S. Titler M. G. (2008). Providing spiritual care to terminally ill older adults. Journal of Gerontological Nursing, 34(7), 8–14. 10.3928/00989134-20080701-08 18649819

[bibr38-14713012251377812] Monteiro A. M. F. Santos R. L. Kimura N. Baptista M. A. T. Dourado M. C. N. (2018). Coping strategies among caregivers of people with Alzheimer disease: A systematic review. Trends in Psychiatry and Psychotherapy, 40(3), 258–268. 10.1590/2237-6089-2017-0065 30304119

[bibr39-14713012251377812] Mumby K. (2023). Supporting spiritual health in dementia using the WELLHEAD toolkit: A ‘story-tale’ from a person with logopenic aphasia. Journal for the Study of Spirituality, 13(1), 46–62. 10.1080/20440243.2023.2187968

[bibr40-14713012251377812] Neugarten B. L. Havighurst R. J. Tobin S. S. (1961). The measurement of life satisfaction. Journal of Gerontology, 16(2), 134–143. 10.1093/geronj/16.2.134 13728508

[bibr41-14713012251377812] Newlin K. Knafl K. Melkus G. D. E. (2002). African-American spirituality: A concept analysis. Advances in Nursing Science, 25(2), 57–70. 10.1097/00012272-200212000-00005 12484641

[bibr42-14713012251377812] Ødbehr L. S. Hauge S. Danbolt L. J. Kvigne K. (2017). Residents’ and caregivers’ views on spiritual care and their understanding of spiritual needs in persons with dementia: A meta-synthesis. Dementia, 16(7), 911–929. 10.1177/1471301215625013 26721285

[bibr43-14713012251377812] O'Malley M. Carter J. Stamou V. LaFontaine J. Oyebode J. Parkes J. (2021). Receiving a diagnosis of young onset dementia: A scoping review of lived experiences. Aging & Mental Health. 10.1080/13607863.2019.1673699 31647324

[bibr45-14713012251377812] Puchalski C. M. King S. D. W. Ferrell B. R. (2018). Spiritual considerations (Vol. 32). Hematology/Oncology Clinics of North America. 10.1016/j.hoc.2018.01.011 29729785

[bibr46-14713012251377812] Radloff L. S. (1977). The CES–D scale: A self-report depression scale for research in the general population. Applied Psychological Measurement, 1(3), 385–401. 10.1177/014662167700100

[bibr47-14713012251377812] Sainz M. James T. Strader U. Gore J. Epps F. (2023). “I didn't know I needed to be still”: Experiences of Black dementia caregivers attending tailored online worship services. Research in Gerontological Nursing, 16(6), 273–282. 10.3928/19404921-20230706-03 37450781

[bibr48-14713012251377812] Salsman J. M. Fitchett G. Merluzzi T. V. Sherman A. C. Park C. L. (2015). Religion, spirituality, and health outcomes in cancer: A case for a meta-analytic investigation. Cancer, 121(21), 3754–3759. 10.1002/cncr.29349 26258400 PMC4618242

[bibr49-14713012251377812] Sebern M. D. Whitlatch C. J. (2007). Dyadic relationship scale: A measure of the impact of the provision and receipt of family care. The Gerontologist, 47(6), 741–751. 10.1093/geront/47.6.741 18192628

[bibr50-14713012251377812] Toivonen K. Charalambous A. Suhonen R. (2023). A caring and living environment that supports the spirituality of older people with dementia: A hermeneutic phenomenological study. International Journal of Nursing Studies, 138(2), Article 104414. 10.1016/j.ijnurstu.2022.104414 36549146

[bibr51-14713012251377812] Tricco A. C. Lillie E. Zarin W. O'Brien K. K. Colquhoun H. Levac D. Moher D. Peters M. D. Horsley T. Weeks L. Hempel S. Akl E. A. Chang C. McGowan J. Stewart L. Hartling L. Aldcroft A. Wilson M. G. Garritty C. Straus S. E. (2018). PRISMA extension for scoping reviews (PRISMA-ScR): Checklist and explanation. Annals of Internal Medicine, 169(7), 467–473. 10.7326/M18-0850 30178033

[bibr52-14713012251377812] Uzun U. Başar S. Saritaş A. (2024). Spiritual needs of family caregivers in palliative care. BMC Palliative Care, 23(1), 256. 10.1186/s12904-024-01589-y 39511622 PMC11542245

[bibr53-14713012251377812] Wolverson E. Backhouse T. Barbosa A. Burnand A. Eriksen S. de Paiva A. F. Dening K. H. Koopmans R. Mabire J. B. Sabatini S. van der Steen J. T. (2024). Psychosocial interventions to provide spiritual support for people with dementia: A scoping review protocol. Open Science Framework. 10.17605/OSF.IO/RWJD5

[bibr54-14713012251377812] World Health Organization . (2002, June 1). Palliative care. https://www.who.int/europe/news-room/fact-sheets/item/palliative-care (Accessed 9 July 2025).

[bibr55-14713012251377812] World Health Organization . (2012). Dementia: A public health priority. Available at: https://www.who.int/publications/i/item/dementia-a-public-health-priority (Accessed 5 March 2025).

[bibr56-14713012251377812] Wu L. F. Koo M. (2016). Randomized controlled trial of a six-week spiritual reminiscence intervention on hope, life satisfaction, and spiritual well-being in elderly with mild and moderate dementia. International Journal of Geriatric Psychiatry, 31(2), 120–127. 10.1002/gps.4300 25965388

[bibr57-14713012251377812] Yamamoto-Mitani N. Sugishita C. Ishigaki K. Hasegawa K. Maekawa N. Kuniyoshi M. Hayashi K. (2001). Development of instruments to measure appraisal of care among Japanese family caregivers of the elderly. Scholarly Inquiry for Nursing Practice, 15(2), 113–135. 10.1891/0889-7182.15.2.113 11695490

[bibr58-14713012251377812] Zarit S. H. Reever K. E. Bach-Peterson J. (1980). Relatives of the impaired elderly: Correlates of feelings of burden. The Gerontologist, 20(6), 649–655. 10.1093/geront/20.6.649 7203086

