# Supplementary material for: Spiritual Support for People Affected by Dementia: A Scoping Review
Source: Dementia (London). 2025 Sep 9;25(7):1674–99. doi: 10.1177/14713012251377812 (PMC13569916; doi:10.1177/14713012251377812)
Supplement: Supplementary file 1 [file sj-pdf-1-dem-10.1177_14713012251377812.pdf]

Supplement A: Search Terms

Search terms for electronic databases.	
Spirituality	TI (spiritual OR spirituality OR spirit OR religion OR religiosity OR religious OR "spiritual needs" OR "holistic care" OR values OR beliefs OR traditions OR existential* OR "Theologic Prayer" OR Namaste OR Faith OR Faiths OR Minister* OR "Pastor*OR Rabbi*" OR Decon* OR Imam OR Imams OR Priest* OR Cleric* OR Clergy* OR Church* OR Chaplain* OR Christian* OR Catholic* OR Lutheran* OR Protestant* OR Anglican* OR Episcopal* OR Orthodox* OR Baptist* OR Methodis* OR Pentacost* OR Jew OR Jews OR Jewish OR Judai* OR Islam* OR Muslim* OR Hindu OR Hindus OR Hinduism OR Buddhis* OR Sikh* OR Mormon*) OR AB (spiritual OR spirituality OR spirit OR religion OR religiosity OR religious OR "spiritual needs" OR "holistic care" OR values OR beliefs OR traditions OR existential* OR "Theologic Prayer" OR Namaste OR Faith OR Faiths OR Minister* OR "Pastor*OR Rabbi*" OR Decon* OR Imam OR Imams OR Priest* OR Cleric* OR Clergy* OR Church* OR Chaplain* OR Christian* OR Catholic* OR Lutheran* OR Protestant* OR Anglican* OR Episcopal* OR Orthodox* OR Baptist* OR Methodis* OR Pentacost* OR Jew OR Jews OR Jewish OR Judai* OR Islam* OR Muslim* OR Hindu OR Hindus OR Hinduism OR Buddhis* OR Sikh* OR Mormon*) OR SU (spiritual OR spirituality OR spirit OR religion OR religiosity OR religious OR "spiritual needs" OR "holistic care" OR values OR beliefs OR traditions OR existential* OR "Theologic Prayer" OR Faith OR Faiths OR Minister* OR "Pastor*OR Rabbi*" OR Decon* OR Imam OR Imams OR Priest* OR Cleric* OR Clergy* OR Church* OR Chaplain* OR Christian* OR Catholic* OR Lutheran* OR Protestant* OR Anglican* OR Episcopal* OR Orthodox* OR Baptist* OR Methodis* OR Pentacost* OR Jew OR Jews OR Jewish OR Judai* OR Islam* OR Muslim*

	OR Hindu OR Hindus OR Hinduism OR Buddhis* OR Sikh* OR Mormon*)
Dementia	TI (dementia OR Alzheimers OR "Cognitive decline" OR "Cognitive impair*" OR Memory OR "Advanced Dementia" OR "Severe Dementia" OR "End stage Dementia" OR "Advanced Alzheimer" OR Severe OR Alzheimer OR "End stage Alzheimer") OR AB (dementia OR Alzheimers OR "Cognitive decline" OR "Cognitive impair*" OR Memory OR "Advanced Dementia" OR "Severe Dementia" OR "End stage Dementia" OR "Advanced Alzheimer" OR Severe OR Alzheimer OR "End stage Alzheimer") OR SU (dementia OR Alzheimers OR "Cognitive decline" OR "Cognitive impair*" OR Memory OR "Advanced Dementia" OR "Severe Dementia" OR "End stage Dementia" OR "Advanced Alzheimer" OR Severe OR Alzheimer OR "End stage Alzheimer")
Psychosocial	TI ('psychosocial*' OR 'non pharmacological*' OR 'non drug related') OR AB ('psychosocial*' OR 'non pharmacological*' OR 'non drug related') OR SU ('psychosocial*' OR 'non pharmacological*' OR 'non drug related')
Intervention	TI (intervention* OR therap* OR treatment* OR approach* OR support) OR AB (intervention* OR therap* OR treatment* OR approach* OR support) OR SU (intervention* OR therap* OR treatment* OR approach* OR support)

TI= TITLE; AB= ABSTRACT

Supplement B

Table S1. Appraisal of included empirical primary studies with the Mixed methods appraisal tool (MMAT)

**Study**	**Criteria met of 5***					**Total %met** versus unmet or uncertain **	**Criteria study type***	**Comments and justification for any downgrades**
	1	2	3	4	5			
Burke et al. (2018)***	+	-	+	+	+	80%	Qualitative part (1.1-1.5)	1.2 choice methods not justified
	+	?	+	?	+	60%<br><br>[100% without uncertain]	Quantitative descriptive part (4.1-4.5)	4.2 population sample drawn from not clearly described<br>4.4 details on efforts to minimise bias not reported
Epps et al. (2020)	+	+	+	+	+	100%	Qualitative (1.1-1.5)	No comments
Epps et al. (2021)	+	+	+	+	+	100%	Qualitative (1.1-1.5)	No comments
Glueckauf et al. (2009)	-	+	-	?	-	20%<br><br>[40% without uncertain]	Pre-post evaluation (3.1-3.5)	3.1 & 3.4 poor recruitment from one site only
Haufe et al. (2024a)	+	+	+	+	+	100%	Participatory research (1.1-1.5)	No comments
Haufe et al. (2024b)	+	+	+	+	+	100%	Participatory research (1.1-1.5)	No comments
Kirkland et al. (2014)	+	+	+	?	+	80%<br><br>[100% without uncertain]	Mixed methods (5.1-5.5)	5.4 DCM not straight forward. Spirituality could be expressed in internalisation, e.g. that may be interpreted as apathy or sleep

Mackinlay & Trevitt (2010)	-	+	-	+	-	40%	Qualitative (1.1-1.5)	1.1 design not most suitable for questions related to impact / effect assessment<br>1.3 unclear how rich the data were and how the results were derived from it<br>1.5 aim does not relate to discussion, methods and results
Mumby (2023)	?	?	+	+	+	60%<br><br>[100% without uncertain]	Qualitative (1.1-1.5)	1.1 and 1.2 research question not clearly stated
Sainz et al. (2023)	+	+	+	?	+	80%<br><br>[100% without uncertain]	Mixed methods (5.1-5.5)	5.4 any divergences between quantitative and qualitative were not reported / discussed
Toivonen et al. (2022)	+	+	+	?	+	80%<br><br>[100% without uncertain]	Qualitative (1.1-1.5)	1.4 limited detail on assessment of capacity
Wu & Koo (2015)	?	+	+	-	?	40%<br><br>[80% without uncertain]	RCT (2.1-2.5)	2.1 randomisation based on random numbers is unclear<br>2.4 self reported outcomes<br>2.5 adherence to intervention not reported no process evaluation and nothing in the limitations

* The number of green boxes with + indicates number of criteria met. The number of orange boxes with - indicates the criteria that were not met and grey boxes with question marks indicate “can’t tell;” both are explained in the last column.

** Some prescribed questions of the MMAT were not appropriate for the design of the research. Therefore we decided that for the overall rating it would be unfair to downgrade if it was uncertain whether a criterion was met (indicated with a ? and grey colour).

*** Burke et al. (2018) did not report their study design. Therefore, the qualitative and quantitative components were rated separately, as the study did not claim to be mixed methods and may not have sought to integrate methods.

MMAT criteria for qualitative studies:

1.1. Is the qualitative approach appropriate to answer the research question?

1.2. Are the qualitative data collection methods adequate to address the research question?

1.3. Are the findings adequately derived from the data?

1.4. Is the interpretation of results sufficiently substantiated by data?

1.5. Is there coherence between qualitative data sources, collection, analysis and interpretation?

MMAT criteria for quantitative randomised controlled trials

2.1 Is randomization appropriately performed?

2.2 Are the groups comparable at baseline?

2.3 Are there complete outcome data?

2.4 Are outcome assessors blinded to the intervention provided?

2.5 Did the participants adhere to the assigned intervention?

MMAT criteria for non-randomized studies

3.1. Are the participants representative of the target group?

3.2. Are measurements appropriate regarding both outcome and intervention (or exposure)?

3.3. Are there complete outcome data?

3.4. Are the cofounders accounted for in the design and analysis?

3.5. During the study period, is the intervention administered (or exposure occurred) as intended?

MMAT criteria for quantitative descriptive studies:

4.1. Is the sampling strategy relevant to address the research question?

4.2. Is the sample representative of the target population?

4.3. Are the measurements appropriate?

4.4. Is the risk of nonresponse bias low?

4.5. Is the statistical analysis appropriate to answer the research question?

MMAT criteria for mixed-methods studies:

5.1. Is there an adequate rationale for using a mixed methods design to address the research question?

5.2. Are the different components of the study effectively integrated to answer the research question?

5.3. Are the outputs of the integration of qualitative and quantitative components adequately interpreted?

5.4. Are divergences and inconsistencies between quantitative and qualitative results adequately addressed?

5.5. Do the different components of the study adhere to the quality criteria of each tradition of the methods involved
